# Lockdowns aimed at fighting COVID-19 causing more harm than good in sub-Saharan Africa

**DOI:** 10.11604/pamj.2021.39.102.26023

**Published:** 2021-06-03

**Authors:** Choolwe Muzyamba

**Affiliations:** 1 University College Utrecht, Campusplein 1, 3584 ED Utrecht, the Netherlands,; 2 University of Amsterdam, Politics, Psychology, Law and Economics (PPLE) College, Roetersstraat 11, 1018 WB Amsterdam, the Netherlands

**Keywords:** COVID-19, lockdown, sub-Saharan Africa

## Abstract

The emergency of COVID-19 has forced many sub-Saharan African (SSA) governments to lockdown countries. This meant minimizing interaction between individuals through actions such as closure of schools, restaurants, bars, and imposing restrictions on movements and events. Supporters of lockdowns argue that lockdowns are useful for slowing down the spread of the disease, preventing the health care systems from potential collapse and preventing deaths. While they are well-intended, these arguments in support of lockdowns are out of touch with reality in SSA. The socioeconomic, psychological and political impact of lockdowns may be much larger than its benefits. Total population lock-downs in the context of SSA seem to be unhelpful especially given that the population at risk is a small identifiable demographic. A more useful approach would be to isolate, focus and direct available care to the most at risk population in context-specific ways and carefully open up the countries.

## Commentary

The onset of the Corona Virus Diseases of 2019 (COVID-19) pandemic has led to debates on the relevance of country-wide lockdowns as tools for responding to this pandemic. This commentary contributes to this debate by focusing on sub-Saharan Africa (SSA). Thus the aim here is to interrogate the relevance of total country lockdowns in responding to the COVID-19 pandemic in SSA. A search of existing literature in academic journals was done in order to build the premise of the commentary. The search included capturing publications that focused on COVID-19 with relevance to SSA; through this process, 8 publications were identified and included in the study.

The emergency of COVID-19 took SSA by surprise. Countries in the region did not seem prepared for the arrival of a pandemic of this magnitude. Seeing the effects of the disease in some parts of Asia and Europe, many wondered what damage it could inflict on SSA, a region still experiencing the negative impacts of HIV/AIDS and Ebola pandemics. The billionaire philanthropist Bill Gates warned as early as February that Africa would be the worst-hit region of the world [1].

While there has been support for lockdowns by several scholars who posit that lockdowns are useful for slowing down the spread of the disease, preventing the health care systems from potentialcollapseand preventing deaths [2-5]. While they are well-intended and useful, these arguments in support of lockdowns are in some cases out of touch with reality in most parts of SSA.

Firstly, most (but certainly not all) living conditions of people in SSA are not compatible with the concept of social distancing. For example, while there are some exceptions, there are several African cities that are home to slums and other unplanned settlements which means that people in such situations live densely together and have to share essential services such as water sources, and bathrooms with several other people in communities. This means avoiding contact to stop the spread of the virus is practically impossible under such living conditions.

Some scholars also point out that the economic situation at household level in the region is at odds with the demands of a lockdown [6]. Lockdowns require people to stock-up food and other essential products. However, most households in SSA have limited savings and mostly live hand-to-mouth with the majority of them working in informal sectors where job-security is mostly nonexistent. Stifling the possibility of people´s daily money-generating errands via the given lockdowns makes it difficult for households to stock-up and let alone realize the possibility of three decent meals a day. Some families have already expressed fears that they might die out of hunger before the virus visits their households [7]. To these families, the certainty of starvation is less daunting than the lottery of COVID-19. All in all, lockdowns are set to rise the already critical poverty levels in the region, and without state-sanctioned social protection measures, the situation may soon become dire. Lockdowns seemto have been initiated and implemented obliviousto these realities.

The argument that lockdowns relieves pressure on the health care system is disingenuous. Firstly, not only is this argument wrong in the African context, it is also an inherently flawed and highly questionable epidemiological preposition. Total lockdowns seem beneficial in situations where the virus presents an imminent threat to a large proportion of the population. This is not the case for COVID-19. The most-at-risk population has been identified to be the elderly and adults with some pre-existing illnesses such as diabetes, cardiovascular disease, respiratory complications and compromised immunity systems. By focusing on the entire population, a lockdown seems to take away specific attention from this population at risk. This is a sub-population that needs specific attentive care, but its needs are obscured by an attempt to appeal to a much larger but relatively low-risk population via lockdowns. This is a relatively small proportion of the population which can be isolated and provided with the best available care to prevent them from getting sick in the first place. A general focus on the entire population via total lockdowns takes away focus, money, specific attention and care that should go to this risky group. It is this lockdown approach that is likely to increase the number of those who actually get seriously ill and overwhelm the health care system in the region.

The lockdown also presents some psychological ramifications which may have long term effects on the region. Armitage and Nellums (2020) for example show that COVID-19-related lockdowns led to severe anxiety and depression, especially among the marginalized groups [8,9]. This is because lockdowns curtail channels of constant social interaction thereby leading to feelings of loneliness and social isolation which are a good breeding ground for anxiety and depression [8]. Most African cultures thrive on daily social contact and interaction, thus these measures at such proportion present even greater risks to Africans in whose culture grand social isolation is alien. Burgess (2020) also emphasizes that the marginalized people within Africa face daunting mental health problems given the lack of economic security. It therefore seems plausible that lockdowns have only worsened the situation [9].

Politically, it seems some politicians are taking advantage of the pandemic to seize more power and further their acts of political repressions. There are already reports in which security personnel in countries like South Africa, Zambia and Kenya have been deployed on the streets in an effort to protect the ends of the lockdown but are simultaneously engaged in gross violations of human rights. Reports of state-sanctioned violence against the marginalized have been reported in various parts of Africa.

**Availability of data and materials:** all materials relating to this study are provided.

**Funding:** the study was self-funded.

In sum, in the context of SSA, the socioeconomic, psychological and political impact of lockdowns may be much larger than that of its benefits. Although, total population lock-downs can play a role in slowing down the spread of the virus, they however present a much higher cost to society. In the context of SSA, lock-downs seem to be unhelpful especially given that the population at risk is a small identifiable demographic. A more useful approach would be to isolate, focus and direct available care to the most at risk population in context-specific ways and carefully open up the countries.
